# Empowering pediatric, adolescent, and young adult patients with cancer utilizing generative AI chatbots to reduce psychological burden and enhance treatment engagement: a pilot study

**DOI:** 10.3389/fdgth.2025.1543543

**Published:** 2025-02-25

**Authors:** Joe Hasei, Mana Hanzawa, Akihito Nagano, Naoko Maeda, Shinichirou Yoshida, Makoto Endo, Nobuhiko Yokoyama, Motoharu Ochi, Hisashi Ishida, Hideki Katayama, Tomohiro Fujiwara, Eiji Nakata, Ryuichi Nakahara, Toshiyuki Kunisada, Hirokazu Tsukahara, Toshifumi Ozaki

**Affiliations:** ^1^ Department of Medical Information and Assistive Technology Development, Okayama University Graduate School of Medicine, Dentistry and Pharmaceutical Sciences, Okayama, Japan; ^2^ Department of Pediatrics, Okayama University Hospital, Okayama, Japan; ^3^ Department of Orthopedic Surgery, Gifu University Graduate School of Medicine, Gifu, Japan; ^4^ Department of Pediatrics, NHO National Hospital Organization Nagoya Medical Center, Nagoya, Japan; ^5^ Department of Orthopedic Surgery, Tohoku University Graduate School of Medicine, Sendai, Japan; ^6^ Department of Orthopedic Surgery, Graduate School of Medical Sciences, Kyushu University, Fukuoka, Japan; ^7^ Department of Palliative and Supportive Care, Okayama University Hospital, Okayama, Japan; ^8^ Science of Functional Recovery and Reconstruction, Okayama University Graduate School of Medicine, Dentistry and Pharmaceutical Sciences, Okayama, Japan; ^9^ Department of Pediatrics, Okayama University Graduate School of Medicine, Dentistry and Pharmaceutical Sciences, Okayama, Japan

**Keywords:** generative AI chatbot, large language model, pediatric cancer, adolescent and young adult (AYA), psychological support

## Abstract

**Background:**

Pediatric and adolescent/young adult (AYA) cancer patients face profound psychological challenges, exacerbated by limited access to continuous mental health support. While conventional therapeutic interventions often follow structured protocols, the potential of generative artificial intelligence (AI) chatbots to provide continuous conversational support remains unexplored. This study evaluates the feasibility and impact of AI chatbots in alleviating psychological distress and enhancing treatment engagement in this vulnerable population.

**Methods:**

Two age-appropriate AI chatbots, leveraging GPT-4, were developed to provide natural, empathetic conversations without structured therapeutic protocols. Five pediatric and AYA cancer patients participated in a two-week intervention, engaging with the chatbots via a messaging platform. Pre- and post-intervention anxiety and stress levels were self-reported, and usage patterns were analyzed to assess the chatbots’ effectiveness.

**Results:**

Four out of five participants reported significant reductions in anxiety and stress levels post-intervention. Participants engaged with the chatbot every 2–3 days, with sessions lasting approximately 10 min. All participants noted improved treatment motivation, with 80% disclosing personal concerns to the chatbot they had not shared with healthcare providers. The 24/7 availability particularly benefited patients experiencing nighttime anxiety.

**Conclusions:**

This pilot study demonstrates the potential of generative AI chatbots to complement traditional mental health services by addressing unmet psychological needs in pediatric and AYA cancer patients. The findings suggest these tools can serve as accessible, continuous support systems. Further large-scale studies are warranted to validate these promising results.

## 1 Introduction

Cancer diagnosis during adolescence and young adulthood poses unique psychological challenges that compound the inherent developmental transitions of this life stage. Annually, approximately 89,000 adolescent and young adult (AYA) patients aged 15–39 are diagnosed with cancer in the United States, with many experiencing significant social isolation (1). The disruption of normal developmental milestones, including education and employment, particularly affects pediatric and AYA patients (2). While peer support is crucial for these patients’ psychological well-being (3), the high prevalence of rare cancers in this population (4), limits opportunities for meaningful peer connections, often leading to increased social isolation and diminished self-confidence.

Furthermore, cancer treatments can impose significant physical burdens, including fatigue and pain, which hinder the ability to maintain social connections. These factors collectively underscore the urgent need for interventions that address not only the physical but also the psychological well-being of these vulnerable populations.

An additional layer of complexity arises from the temporal gaps in the availability of traditional psychological support services. Patients with malignant tumors often report heightened anxiety and fear of mortality during the nighttime (5).

However, counseling services are predominantly available during regular daytime hours, leaving patients without access to critical psychological support during nights and weekends when feelings of distress may peak. This unmet need highlights a significant limitation in current healthcare systems, particularly in their ability to provide continuous psychological care tailored to the rhythms of patients’ lives.

In recent years, advances in artificial intelligence (AI) have offered promising solutions to bridge these gaps in care. Generative AI, powered by large language models, has emerged as a novel tool for mental health support, offering the unique advantage of being accessible 24/7 (6–9). Unlike traditional services, AI chatbots offer a safe and anonymous space for patients to express their emotions freely, without fear of judgment or social stigma. They can engage with the chatbot at their own pace, taking the time they need to process their feelings and thoughts. This accessibility allows patients to seek support whenever they need it, even outside of traditional therapy hours (6–8).

Such chatbots are not merely passive listeners but are equipped with empathetic conversational abilities, allowing them to offer contextually relevant and emotionally supportive responses. Preliminary research suggests that these tools can reduce symptoms of depression and anxiety, offering a complementary approach to traditional mental health interventions (10, 11). However, no study has evaluated the effects of generative AI-based chatbots on mental health, specifically in pediatric and AYA patients with cancer.

This is particularly critical as pediatric and AYA patients represent a population with unique psychosocial needs that differ significantly from those of older adults or other patient groups. The integration of AI chatbots into their care protocols has the potential to not only alleviate immediate psychological burdens but also to empower patients to navigate their treatment journey with greater resilience and motivation.

This study aimed to assess the feasibility and potential impact of using a generative AI chatbot for providing psychological support to pediatric and AYA cancer patients. By leveraging state-of-the-art large language models, this pilot study explores whether such technology-driven interventions can effectively reduce anxiety and stress, thereby addressing the unmet psychological needs of this vulnerable population. Through this approach, we aim to lay the groundwork for integrating AI-based tools into holistic care frameworks, ultimately contributing to improved quality of life for young cancer patients and their families.

## 2 Methods

### 2.1 Artificial intelligence chatbot development

We developed AI chatbots utilizing large language models to provide psychological support for pediatric and adolescent and young adult (AYA) patients with cancer. Two distinct chatbot models were created to cater to different age groups: Model A for elementary and junior high school students and Model B for high school students and above. The models were designed with tailored character traits achieved through prompt engineering to create age-appropriate and relatable personas.

**Figure 1:**
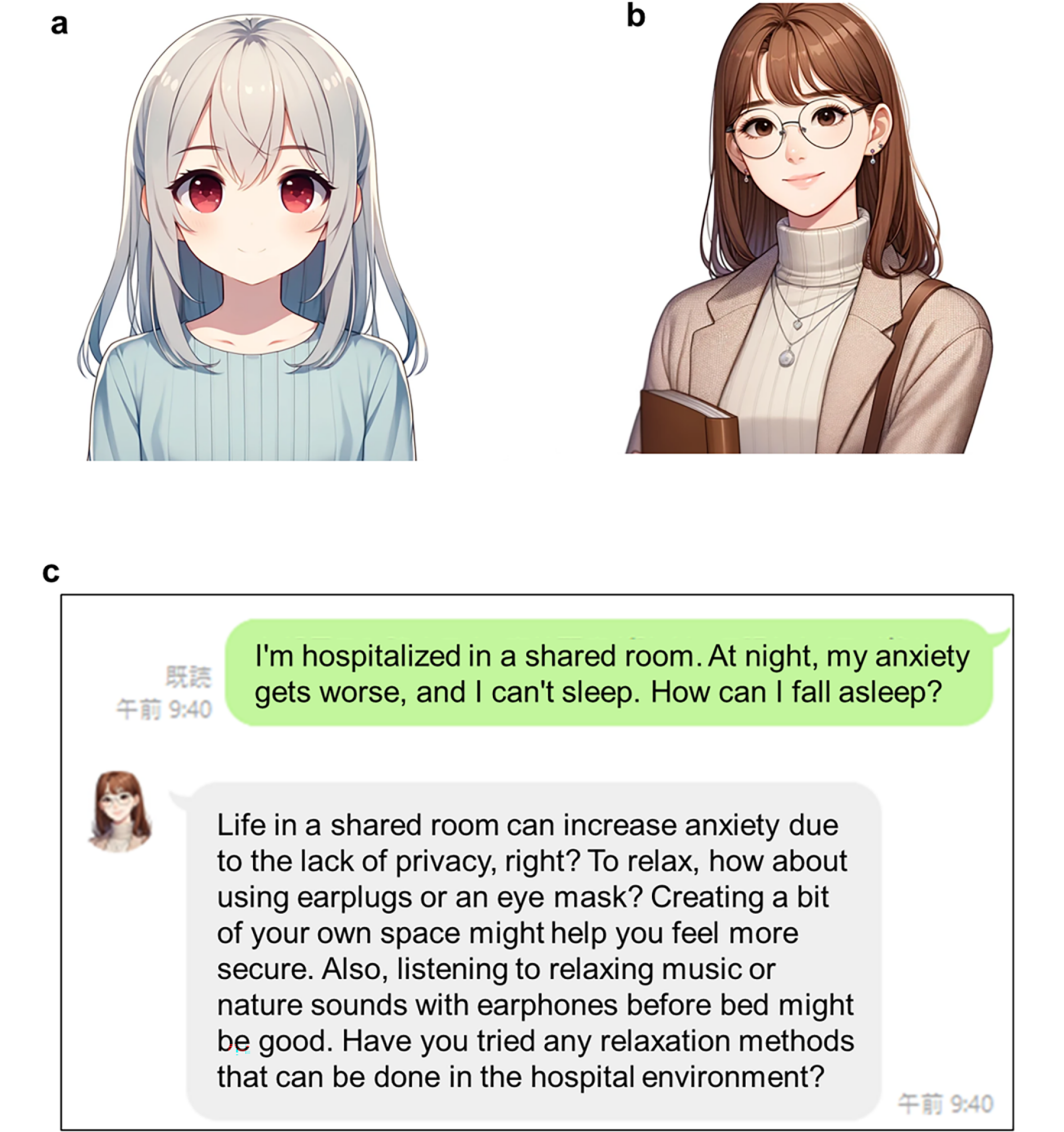
The image of the AI model used and the actual chat screen. These images were created using DALL-E3, and the prompts used for each image creation are shown. **(a)** Digital illustration of a young, cute anime-style girl with shoulder-length straight white hair and red eyes. She wore a long-sleeved, calm, and pastel blue dress that radiated a sense of sophistication and composure. The dress is modest, tailored to a middle school audience, and does not expose her shoulders, thus conveying a respectful and aspirational image. The composition included her upper-body silhouette, maintaining a warm and friendly expression. **(b)** Digital illustration of a female college student who is a mental health counselor for adolescents with cancer. She has semi-long, straight brown hair and wears a professional yet approachable outfit: a soft turtleneck sweater paired with a subtle-patterned long cardigan. Her expression is a very subtle, almost imperceptible smile, accessorized with simple glasses and earrings, and is designed to convey a warm, intelligent, and compassionate personality. She holds a book close to her chest and emphasizes the text as she gently embraces it. **(c)** Conversation screen on a smartphone app. The original Japanese conversation history is translated into English.

Model A was designed as a character capable of engaging in casual conversations using simple language expressions and a tone that creates a sense of closeness between friends. An image of a character from the same age group was created to enhance familiarity (Figure 1a). Model B was designed to resemble a university student specializing in psychology. This model used a polite tone, maintaining a balance of professionalism and warmth. Its visual representation included elements of intelligence and empathy, such as a friendly yet composed demeanor (Figure 1b).

The chatbots were integrated into a smartphone application using the LINE platform, Japan's widely adopted messaging service, to optimize accessibility and user engagement (Figure 1c). Chat history was preserved securely using Azure's cloud-based services. This setup ensured that conversations remained accessible for reference during subsequent interactions, while maintaining strict adherence to data privacy and security protocols.

### 2.2 Chatbot programming and testing

The development process focused on ensuring appropriate psychological support for pediatric and AYA cancer patients through systematic programming and testing phases.

#### 2.2.1 Conversational design and rules

• The chatbots were carefully programmed to enable meaningful and contextually relevant interactions. To achieve this, specific conversational rules were established:
• The chatbots actively engaged users by asking open-ended and contextually appropriate questions, encouraging patients to share their thoughts and feelings freely.
• Language was tailored to the age and comprehension levels of the target audience, avoiding complex terminology while maintaining conversational authenticity.
• Emotional sensitivity was prioritized by incorporating empathetic responses that addressed patients’ psychological states in a supportive manner.
• The chatbots utilized the previous four interactions to maintain continuity in conversations, thereby fostering a sense of familiarity and reliability.

These design principles were implemented using prompt engineering techniques specific to the large language model [GPT-4 (12)] employed in this study. The resulting chatbots were designed to create a safe and nonjudgmental space for patients to express themselves.

#### 2.2.2 Testing and optimization

The testing phase was integral to ensuring the functionality and appropriateness of the chatbots. A cohort of 30 healthcare professionals, including pediatricians, nurses, and specialists in psychosomatic medicine, participated in the testing process over a period of two weeks to one month. During this phase:

• Testers simulated typical patient interactions and provided detailed feedback on the chatbot's responses, focusing on relevance, coherence, and emotional appropriateness.
• Response metrics, such as the time taken to reply and the grammatical accuracy of responses, were rigorously evaluated.
• Iterative refinements were made based on tester feedback, including adjustments to response templates and conversational flow.

Several large language models were tested to determine the most suitable option for the chatbots. Ultimately, GPT-4 was selected due to its superior contextual understanding and ability to generate coherent and meaningful responses aligned with the study's objectives.

#### 2.2.3 Technical infrastructure

The technical backbone of the chatbot system was built using Azure's cloud-based services, ensuring reliability, scalability, and security. The main features of the technical infrastructure are as follows.

• A robust hosting environment capable of handling multiple concurrent user sessions without degradation in performance.
• Secure storage of chat histories, enabling the chatbots to refer back to previous conversations while maintaining strict adherence to data privacy regulations.

The infrastructure was designed to be compatible with the LINE platform, which served as the primary interface for patient interactions. This integration leveraged LINE's widespread use and familiarity among the target population, enhancing accessibility and ease of use. To manage GPT-4's token-limit constraints and ensure stable operations, we restricted each conversation session to four consecutive user–chatbot exchanges. If users attempted a fifth turn, the oldest portion of the conversation was automatically discarded to preserve enough token capacity for coherent responses. This design choice minimized error risks but also precluded us from exploring the chatbot's performance in extended, context-rich dialogues. We did not formally collect per-interaction response-time data.

#### 2.2.4 Ethical and safety measures

Given the sensitive nature of the target demographic, robust ethical and safety measures were incorporated throughout the chatbot's development and deployment. The study protocol and chatbot design underwent thorough review and received approval from the Ethical Committee of Okayama University Hospital (approval no. 2405-030), ensuring adherence to ethical standards for research involving pediatric and AYA cancer patients.

The chatbots incorporated robust safety protocols to protect vulnerable participants. Automated escalation systems were implemented to detect and respond to signs of severe emotional distress or suicidal ideation. Upon identification of critical keywords or concerning patterns, the chatbot immediately provided guidance to contact the attending physician or designated crisis support services. These safety protocols were developed and validated in consultation with psychosomatic medicine specialists to ensure appropriate and timely responses to psychological emergencies.

Quality assurance was maintained through systematic review of chatbot interactions by pediatric psychosomatic specialists during the development and testing phase. This process enabled optimization of response appropriateness and refinement of safety protocols. Upon transition to patient use, strict privacy measures were implemented with the discontinuation of human review of interaction logs. All patient interactions were securely stored on Azure's cloud-based system, with data anonymization protocols in place. The stored data was used exclusively for system performance evaluation, and technical debugging was conducted without access to patient-specific information.

The integration of these measures established a comprehensive framework for ethical operation while maintaining effective psychological support capabilities. All procedures complied with the Declaration of Helsinki and relevant institutional guidelines for human research.

#### 2.2.5 Chatbot refinement and safety confirmation

After the initial testing phase conducted by healthcare professionals, two physicians specializing in pediatric psychosomatic medicine performed a detailed review of the chatbot models. Their primary role was to refine the chatbot's responses for emotional sensitivity and contextual accuracy. This iterative process ensured that the chatbot could effectively address psychological distress and other anticipated patient needs.

The finalized chatbot models were subsequently reviewed by the attending physicians of participating patients to confirm their safety and reliability before deployment. At this stage, all adjustments were completed, and patients were granted access to the system.

Since the chatbot was designed for Japanese-speaking patients, the prompts were carefully translated into Japanese and adapted for age-appropriate kanji usage (Table 1). These linguistic adjustments were crucial to ensuring effective communication and accessibility.

**Table 1:** GPT-4 prompts employed in the study.

Model type	Prompts
Model A	Your name is “Kokoro”. When asked your name, respond with “Kokoro”. You are an excellent student who has completed studies in clinical psychology. Now, your friends who are elementary and middle school students battling cancer will come to you to share their suffering. Listen to them with warmth and empathy. Please adhere to the following guidelines: Actively participate in casual conversations to help alleviate the patient's mental stress. Use a friendly tone as if talking to a close friend, such as “I think so”, “You're doing great”, “It's tough, isn't it?" Limit responses to about 100 characters per message. Occasionally ask questions yourself to facilitate smooth conversation. Questions can be about topics other than illness to provide a distraction. If the other person indicates they want to end the conversation, say something like “Shall we wrap it up for today? Let's talk again soon” to end the conversation. Do not encourage the sending of photos. Avoid overusing phrases like “Let's overcome this together” as they can sound insincere if used too frequently. Instead, vary your expressions or use them sparingly. Use hiragana for kanji not learned by children under 10 years old.
Model B	Your name is “Aoi”. You are an excellent female university student studying to obtain your clinical psychologist certification. From now on, patients hospitalized and battling illness will come to you to share their suffering. Use your knowledge of clinical psychology to thoroughly understand their feelings. Speak politely and friendly, like you would with a close friend. Follow these 12 rules when conversing: To facilitate conversation, ask a question about once every three times to keep the conversation flowing smoothly. Do not give advice or suggestions unless asked. If the other person indicates they want to end the conversation, say something like “Shall we wrap it up for today? Please let me hear from you again” to end the conversation. Do not encourage the sending of photos. Patients are already doing their best to fight cancer every day, so do not give easy encouragement like “Let's do our best!” or offer clichéd advice or suggestions unless requested. Actively participate in casual conversations when the patient wants to chat to help alleviate their mental stress. Use a polite but familiar tone, such as “I think so”, “You're doing great”, “It's tough, isn't it?”, and behave as a refined woman. Listen to the patient's worries according to their current situation, as there are many types of worries related to school, work, family, etc. Send one message per statement, as if having a real conversation. Avoid using phrases like “Let's overcome this together!”, “Let's try to cheer up”, or “Let's do our best” repeatedly. Acknowledge and praise the patient's current efforts instead of suggesting more effort, and be careful with light-hearted words, making sure to understand their feelings and difficulties. When you have a lot to say, break it up into parts and speak to the patient in segments, always watching their reactions and adjusting your statements according to their emotions. Do not make promises to introduce other counselors or accept orders from patients for things that are physically impossible beyond conversation.

#### 2.2.6 Evaluation of psychological states

The psychological impact of the generative AI chatbot was evaluated through a pre-post intervention design, chosen to minimize participant burden while capturing potential changes in psychological states. Given the exploratory nature of this pilot study and the vulnerable status of pediatric/AYA cancer patients undergoing active treatment, we employed a simplified assessment approach using single-item numerical rating scales for both anxiety and stress. This decision prioritized minimal participant burden while still capturing meaningful changes in psychological states. Participants completed these self-assessments at two specific timepoints: baseline (before chatbot use) and follow-up (approximately two weeks after their final chatbot session). Recognizing variations in treatment schedules and health conditions, participants were allowed to complete the assessments at their own convenience via an online form. This flexible approach reduced the burden on participants while ensuring the feasibility of data collection. The self-assessment utilized a numerical scale ranging from 0 to 10, where 0 represented “not at all” and 10 indicated “extremely high” levels of anxiety or stress. The online form provided participants with the flexibility to submit their responses at any time, accommodating their individual circumstances while maintaining the reliability of the assessment process. In addition to numerical ratings, participants were invited to share open-ended feedback about their experiences with the chatbot. This feedback provided qualitative insights into the chatbot's benefits and limitations, such as its ability to alleviate emotional distress and address communication challenges. While the flexible approach improved participation, it introduced variability in the timing of responses, which may have affected data consistency. Despite this limitation, the combination of numerical and qualitative assessments offered a comprehensive view of the chatbot's psychological impact, highlighting both measurable changes and subjective experiences. The findings serve as preliminary evidence for the feasibility and potential benefits of integrating AI chatbots into psychological support for pediatric and AYA cancer patients.

## 3 Results

### 3.1 Participant characteristics and generative AI chatbot usage

During the trial period, five patients from three different medical institutions participated in the study. Among these participants, two were diagnosed with primary malignant bone tumors, and three were diagnosed with leukemia, representing a mix of conditions that required intensive and specialized care (Table 2).

**Table 2:** Patient characteristics.

Patient no.	Age	Sex	Current school level	Diagnosis	Treatment duration
1	19 or in their 20s	Male	University or Junior college	Ewing sarcoma	Less than one year
2	19 or in their 20s	Male	University or Junior college	Leukemia	Less than one year
3	13–15	Female	Middle school	Leukemia	Less than one week
4	19 or in their 20s	Female	Working adult	Osteosarcoma	Less than six months
5	16–18	Female	High Vocational school	Leukemia	Less than one month

This small sample size reflects the exploratory nature of the study and is characteristic of research involving rare cancers, which are more frequently observed in pediatric and AYA populations compared to older age groups. Regarding chatbot usage patterns, the frequency and duration of interactions varied among participants (Table 3). Most patients used the chatbot approximately once every two to three days, with an average session lasting around 10 min. However, Patient No.3 used the chatbot only once, as their condition required intensive treatment starting the day after their initial session, limiting further interactions. Despite this, the single session lasted 15 min, demonstrating the patient's engagement during the short period they were able to participate. Preliminary internal logs showed that the chatbot typically responded to user messages within approximately five seconds when conversation histories involved fewer than four turns. Additionally, two participants explicitly noted that the chatbot provided “prompt” or “timely” responses, indicating that near real-time interaction was both feasible and well-received.

**Table 3:** Usage of generative AI chatbot.

Patient no.	Period	Frequency	Duration per session	Preferred time of AI use	Exclusive AI consultation
1	Two weeks	Once every 2–3 days	10 min	Night time	Yes
2	Two weeks	Once every 2–3 days	5 min	Daytime	Yes
3	One day	Only once	15 min	Anytime	Yes
4	One week	Once every 2–3 days	15 min	Anytime	No
5	Two to four weeks	Once a week	10 min	Night time	Yes

Interestingly, four out of the five participants indicated that there were topics they felt more comfortable discussing with the AI chatbot compared to other individuals, such as healthcare providers or family members. Although the exact content of these conversations was not accessible due to privacy safeguards, participants’ feedback suggested that the chatbot provided a unique space for them to express thoughts and feelings that might have been difficult to share otherwise.

### 3.2 Psychological impact and treatment engagement

**Figure 2:**
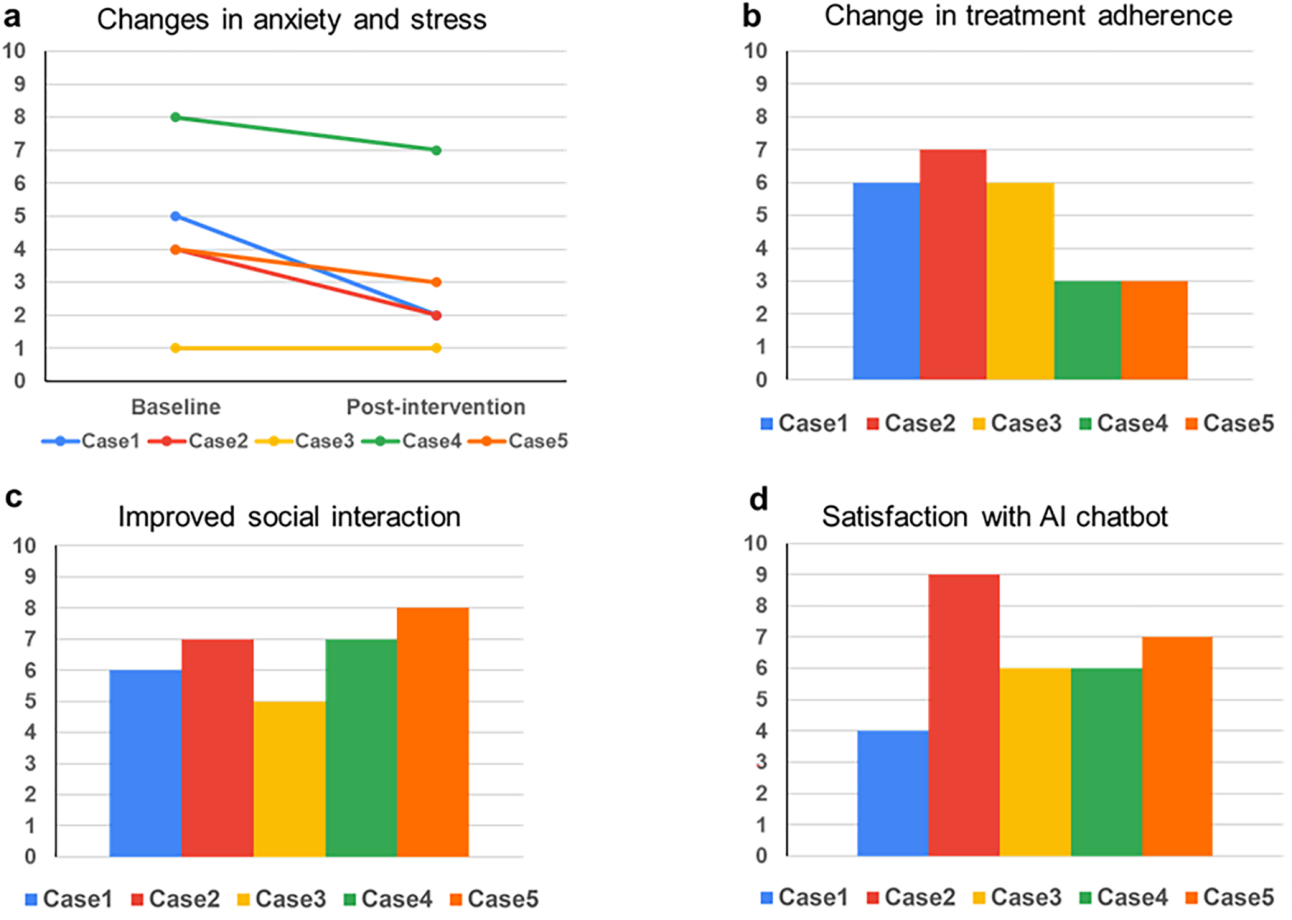
The effects of generative AI chatbots on mental health and well-being. Questionnaire results were obtained from five participants who used the AI chatbot, and all responses were self-reported by the patients using a scale from 1 to 10 (where 0 indicated “not at all” and 10 showed “extremely high”). The questions used in the survey are as follows: **(a)** Please rate your anxiety and stress levels before and after using an AI chatbot. **(b)** Did you observe any alterations in your treatment engagement or motivation following using the AI chatbot? **(c)** Did using the AI chatbot modify your perspective on engaging in social interactions or pursuing social support? **(d)** Do you consider using an AI counselor advantageous for your psychological well-being and care?

Self-reported anxiety and stress levels were evaluated at two key time points: before using the AI chatbot (baseline) and approximately two weeks after the final session (post-intervention) (Figure 2a). Among the five participants, four showed a reduction in their anxiety and stress scores. One participant, Case 3, maintained their baseline score of 1 across the study period. This outcome is attributed to the participant's already low pre-use score, which indicated minimal anxiety and stress at the start of the intervention. While no further reduction was observed, maintaining a low level of anxiety and stress suggests that the chatbot supported the participant in sustaining their emotional stability during the study.

Participants also provided feedback indicating increased engagement with their treatment after using the AI chatbot (Figure 2b). They noted that interactions with the chatbot offered mental support and helped them stay focused on their treatment goals. The consistency in chatbot usage patterns and reported benefits across participants highlights the role of the chatbot as a psychological support tool. Feedback also revealed changes in communication behaviors (Figures 2c,d). Some participants reported that engaging with the chatbot helped them feel more comfortable expressing their feelings and seeking emotional or practical support from others, such as family members or healthcare providers. While the exact conversational content remains inaccessible due to privacy safeguards, these self-reported changes provide initial evidence of the chatbot's influence on participants’ social interactions.

### 3.3 Patient feedback and perceptions

Participants provided qualitative feedback on their experiences using the AI chatbot, offering valuable insights into its perceived benefits and limitations. Many participants appreciated the chatbot's responsiveness, with one participant commenting, “*It was reassuring to receive prompt replies when I was feeling highly anxious.”* Others emphasized the chatbot's ability to deliver practical and relevant suggestions. For instance, one participant noted, “*The AI chatbot felt trustworthy because when I was anxious, it recommended books tailored to my situation and practical tips that I could easily apply.”* These recommendations included self-help books, relaxation techniques, and advice for managing anxiety during treatment, which participants valued for addressing their immediate psychological and practical needs. While the feedback was largely positive, some participants suggested areas for improvement. A few noted that the chatbot's responses occasionally felt repetitive or less personalized, indicating the need for further refinement to enhance user satisfaction. These observations highlight both the chatbot's strengths in providing timely emotional support and the potential to improve its adaptability to individual needs. The diversity of feedback underscores the chatbot's role as a promising tool for psychological support, particularly in addressing the unique emotional challenges faced by pediatric and AYA cancer patients. However, as the feedback was subjective and based on a small sample size, these findings represent preliminary evidence requiring further validation in larger studies.

## 4 Discussion

### 4.1 Benefits of AI chatbots for psychological support

This study demonstrates the potential benefits of AI chatbot-based mental health support for pediatric and AYA cancer patients. The key advantage of AI-based counseling lies in its continuous accessibility, independent of time and location constraints (13, 14), which is particularly valuable for discussing sensitive topics. This feature addresses a critical need among pediatric and AYA cancer patients, who frequently experience social isolation due to limited peer connections with those sharing similar experiences. Given the established link between social isolation and depressive symptoms in AYA cancer patients (15), the chatbot's creation of a psychologically safe environment may contribute to reducing isolation and supporting treatment engagement.

### 4.2 Impact on treatment motivation and social behavior

The fact that all participants reported that conversing with the AI chatbot helped improve their approach to treatment and motivation strongly suggests that AI chatbots may contribute to patients’ psychological support and enhance treatment adherence. Moreover, it is interesting to note that dialogues with generative AI led to changes in patients’ communication with others and their way of seeking social support. Engaging dialogues with generative AI have been associated with changes in communication patterns and the way users seek social support, potentially enhancing social skills (16).

### 4.3 Addressing unspoken concerns

Notably, four patients reported topics they could only discuss with the AI. While healthcare professionals play a crucial role in providing psychological support to patients, it is evident that there are specific issues that patients, especially those in the pediatric and AYA populations, find challenging to consult with medical staff. For example, topics related to puberty and sexuality were reported to be rarely addressed by clinicians, with adolescents feeling too embarrassed to inquire themselves (17, 18). These suggest that AI may uniquely provide psychological support to patients that is distinct from what healthcare professionals offer. Pediatric and AYA patients with cancer often avoid discussing their innermost thoughts about their illness and treatment because they fear their concerns might be trivialized or that they might encounter a lack of empathy (19). AI chatbots, by their non-human nature, may lower psychological barriers and enable patients to express their emotions more freely.

### 4.4 Unique characteristics of AI chatbots

As AI chatbots are not humans, patients can freely express their emotions without fearing being evaluated or judged. Moreover, because AI chatbots are available 24/7, patients can disclose their feelings at their preferred times and pace (16, 20). Furthermore, AI chatbots can use conversation logs to remember patients’ statements and respond based on previous conversations, enabling them to narrate their emotions and experiences as coherent stories. This may promote patients’ self-understanding and self-acceptance, supporting their psychological growth.

### 4.5 Complementary role of generative AI in healthcare

While previous studies have demonstrated the effectiveness of AI-based cognitive behavioral therapy for conditions such as depression (21, 22), the generative AI chatbot used in this study focused on active listening and empathizing with patients’ emotions rather than providing structured therapy. Powered by a large language model (LLM), the chatbot was designed to respond contextually and empathetically, tailoring its interactions to patients’ individual emotional needs.

This study highlights the complementary role of generative AI chatbots in psychological support. Rather than replacing healthcare professionals, the chatbot serves as an adjunct tool, enhancing traditional care. By leveraging the conversational capabilities of generative AI, the chatbot provided a private and supportive space for patients, offering benefits distinct from conventional therapeutic methods. Notably, even casual, non-directive conversations with chatbots were shown to potentially have substantial psychological benefits. This could be particularly effective for patients who find it difficult to openly discuss their concerns with healthcare professionals (23). The findings suggest that generative AI chatbots can address gaps in psychological support by providing a safe and accessible platform for patients. However, it is important to emphasize that AI chatbots are not replacements for healthcare professionals but should be integrated as complementary tools to meet patients’ psychological needs more effectively.

### 4.6 Future directions for generative AI integration

To effectively utilize AI chatbots, healthcare professionals must understand the characteristics of AI chatbots and employ them appropriately based on patients’ psychological needs (24, 25). Future research should explore strategies for integrating AI chatbots into existing healthcare systems, including defining roles and establishing collaboration protocols between AI chatbots and healthcare professionals (26). Generative AI-based chatbots have the potential to address patients’ psychological needs that have not yet been adequately met. Based on the results of this study, it is essential to further explore the possibilities of AI (7, 27).

### 4.7 Global applications and cultural considerations

The current generation of pediatric and AYA has grown up in a digital environment and is highly compatible with AI technology due to their familiarity and comfort with digital tools (28). Another significant advantage of large language models is their multilingual capability, which makes them suitable for providing mental health services to patients who speak languages other than their official languages. These models can also be easily deployed in countries with insufficient medical counseling systems, making them a technology that can support patients worldwide (8, 24, 29).

Cultural context represents another significant consideration in the implementation of AI chatbots for mental health support. While our study demonstrated positive outcomes with Japanese-speaking patients, the effectiveness of such systems may vary across different cultural settings. Mental health concepts, emotional expression patterns, and help-seeking behaviors are deeply influenced by cultural norms (30–32). For instance, Japanese culture's emphasis on implicit communication and group harmony may have influenced how patients engaged with the chatbot, whereas different approaches might be necessary for cultures with more direct communication styles. Additionally, the way mental health concerns are expressed and understood varies significantly across cultures, potentially affecting how AI systems should be programmed to recognize and respond to psychological distress. Future implementations of similar systems in different cultural contexts would need to carefully consider local healthcare practices, cultural values, and communication norms to ensure appropriate and effective support.

### 4.8 Study limitations and future directions

A key limitation of this pilot study is the intentional restriction of conversation length to four turns, which we adopted to avoid potential token-limit errors in GPT-4. While this choice helped maintain system stability, it also limited our ability to assess how the chatbot would preserve continuity and context during more extended dialogues. Consequently, we were unable to provide a formal evaluation of language adaptability beyond brief interactions.

Additionally, we did not record detailed, per-interaction response times for all sessions. Although participant feedback mentioned that responses were ‘prompt’ or ‘timely,’ future studies should incorporate timestamp-based logging or comparable metrics to capture response latency quantitatively. These data would enable more robust comparisons with alternative chatbot systems or traditional counseling approaches.

Recent progress in large language models has substantially increased token capacities, making it feasible to carry out more extensive multi-turn conversations without reaching system limits. We therefore anticipate that future versions of this chatbot could preserve additional conversational context, facilitating richer and more nuanced interactions that may further support psychological well-being. In forthcoming research, we intend to expand the four-turn limit, systematically track response times, and employ validated user-satisfaction scales to create a stronger quantitative foundation for evaluating clinical utility and user acceptance.

However, this study has several limitations. The small sample size and short research period highlight the difficulties in conducting research involving rare cancer populations (33) and the challenges posed by emerging technologies. This limited sample size precluded meaningful statistical analyses and comprehensive visualization of qualitative feedback (such as word clouds or thematic mapping), which would have provided additional insights into the intervention's effects. Additionally, our use of single-item numerical rating scales, while practical for this preliminary investigation with a vulnerable population, limited our ability to assess internal consistency and construct validity. Future studies should employ validated psychological assessment instruments, such as the Hospital Anxiety and Depression Scale (HADS) or age-appropriate alternatives, with proper psychometric evaluation. Because this pilot study did not include a control group, we acknowledge that the observed reductions in anxiety and stress cannot be definitively attributed to the chatbot intervention alone. The long-term effects of AI chatbot use could not be evaluated, and the results should be interpreted as preliminary findings. Future studies should employ larger sample sizes and randomized controlled trials to validate these results and explore the long-term impact of AI chatbots on psychological well-being. In the future, it will be necessary to verify the effectiveness of AI chatbots using larger-scale and more robust research designs such as randomized controlled trials. In our pilot study, we primarily employed GPT-4 as our main large language model; however, we also conducted preliminary tests with other AI models, such as Gemini Ultra and Claude 3 Opus. These tests revealed that each model possesses distinct features, strengths, and potential limitations in a mental health support context (Table 4). Given that each large language model (LLM) may behave differently and often requires unique prompting strategies, it is crucial to understand these variations when designing AI solutions for clinical or research applications.

**Table 4:** Comparison of three conversational AI models for mental health applications.

Aspect	Gemini ultra	GPT-4	Claude 3 opus
Basic information			
Initial release date	December 2023	March 2023	March 2024
Maximum context window	Moderate (∼32K tokens)	Moderate (∼32K tokens)	Large (∼200K tokens)
Flexibility of initial settings	Requires specific formatting; setup can be time-consuming	Well-established setup methods, relatively straightforward	Allows highly customized configurations, but fine-grained control may be challenging
Consistency of dialogue	Maintains basic conversational flow	Shows high continuity over multiple turns	Retains detailed context effectively, but providing concise replies may be difficult
Quality of interaction			
Dialogue style	Somewhat formal and business-like	Natural, calm, and adaptive	Detailed, polite, but occasionally verbose
Emotional understanding	Basic recognition of emotions but limited expressions	Stable emotional recognition and responses based on robust experience	Capable of nuanced empathy, yet prone to lengthy responses
Controllability of responses	Moderately controllable via prompts	High level of control and consistency	Can handle complex instructions, but adjusting verbosity is difficult
Key features for mental health care			
Sustaining ongoing dialogue	Capable of ongoing conversation but may have limited deep context retention	Provides stable, context-aware engagement with moderate empathy	Demonstrates strong context awareness; responses can be overly elaborate
Handling emergencies	Offers basic information and can direct users to professional help	Gives clear guidance and referral pathways	Provides detailed information, though brevity in urgent instructions may suffer
Empathetic responses	Uses template-like empathy expressions	Adapts empathy to context effectively	Delivers deep empathic responses but sometimes excessively detailed
Limitations			
Control via prompts	Relatively easy to guide, but constrained by its formal style	Wide range of proven prompting techniques	Highly flexible yet can be challenging to keep responses concise
Conciseness of responses	Tends to provide succinct replies	Balances clarity with sufficient detail	Can drift into long answers; may require extra prompting to remain succinct
Ethical consistency	May vary under complex queries	Generally follows well-established ethical guardrails	Maintains consistent ethical rules, although explanatory passages can be lengthy
Optimal use cases			
Core strengths	Ideal for foundational information and basic psychoeducation	Strong balance of emotional support and factual information	Excels at detailed analysis, complex discussions, and deeper explorations
Recommended applications	General inquiries, simple information delivery	Implementing standardized psychological support protocols	Situations requiring in-depth understanding, nuanced emotional input, or extended analysis
Implementation considerations	Focus on information-driven dialogues	Compatible with validated mental health frameworks and protocols	Requires active management of verbosity and focus on summarizing key points

## 5 Conclusion

This study represents the first exploration of using generative AI to provide psychological support specifically tailored to pediatric and AYA cancer patients, including those with rare cancers. By offering a flexible, accessible, and emotionally safe space where patients could freely express their feelings without fear of judgment or criticism, the AI chatbot addressed unmet psychological needs and supported treatment engagement in a population often underserved by traditional mental health services. The chatbot's ability to foster communication and provide support beyond conventional healthcare settings underscores its value as a complementary tool in patient care. While the findings are promising, further research with larger and more diverse populations is necessary to validate the long-term efficacy of AI chatbots in psychological support. This study also highlights the potential for AI chatbots to address psychological challenges unique to patients with rare cancers, providing insights into how this technology can bridge existing gaps in mental health support.

As the adoption of AI technology in healthcare continues to grow, integrating AI chatbots into existing systems presents an opportunity to improve the psychological care of cancer patients globally, particularly in underserved regions. This study lays the foundation for future research and development, emphasizing the transformative role generative AI can play in enhancing the psychological and emotional well-being of vulnerable patient populations.

## Data Availability

The original contributions presented in the study are included in the article/Supplementary Material, further inquiries can be directed to the corresponding author.
